# Integrating a life course perspective in work environment and health research: empirical challenges and interdisciplinary opportunities

**DOI:** 10.5271/sjweh.4174

**Published:** 2024-07-01

**Authors:** Ute Bültmann, Karin Broberg, Jenny Selander

**Affiliations:** Department of Health Sciences, Community and Occupational Medicine, University of Groningen, University Medical Center Groningen, Groningen, The Netherlands. [Email: u.bultmann@umcg.nl]; Department of Laboratory Medicine, Lund University, Lund, Sweden. [Email: karin.broberg@ki.se]; Institute of Environmental Medicine, Karolinska Institutet, Stockholm, Sweden. [Email: jenny.selander@ki.se]

A healthy working life is fundamental for individuals and society. To date, increasingly research connects the earlier, pre-working life to later working life experiences and beyond, recognizing that a worker’s health and exposure starts before the working life begins. The research, however, often lacks a fundamental understanding of (i) the underlying mechanisms and pathways accounting for differences in different life stages and (ii) the role of the social environment in shaping working life experiences. By integrating a life course perspective in our research and crossing disciplinary borders in rigorous, collaborative research, we may get a better understanding of the complex and dynamic interplay between work, environment and health.

## A life course perspective for work environment and health research

A life course perspective in work environment and health research emphasizes the importance of prior life experiences, including the environments in which individuals were raised and exposed, their familial and educational backgrounds, and their physical and mental health status before entering the workforce (1, 2). Life course research in different disciplines has been instrumental in developing more robust causal models (3, 4), particularly for understanding developmental health trajectories and socioeconomic health inequalities (eg 5–7).

Adopting an interdisciplinary life course perspective in work environment and health research helps researchers answering questions as to whether and how the timing, duration, intensity, and context of past and present exposures (ie, pre-working, working, and non-working exposures) are associated with later life work and health outcomes. For instance, the ‘exposome paradigm’ is a concept used to describe the sum of occupational and environmental exposures an individual encounters throughout life, and how these exposures impact biology and health (8). In exposome research, a broad range of genetic, biological, chemical, physical, social and lifestyle factors is examined throughout the life course to provide a comprehensive picture of potential risk factors impacting working life health (9). In exposome research and beyond, it is important to examine how the exposure-outcome relationships are shaped by specific social, cultural and historical contexts (2). The conceptual framework of the ‘Social Exposome’ may help to integrate the social environment in conjunction with the physical environment into the exposome concept (10). Moreover, focusing on both historical and contemporary contexts is essential not only for advancing research but also for informing policy and practice, for example by identifying entry points for interventions.

## Exposures during the life course

**Figure 1:**
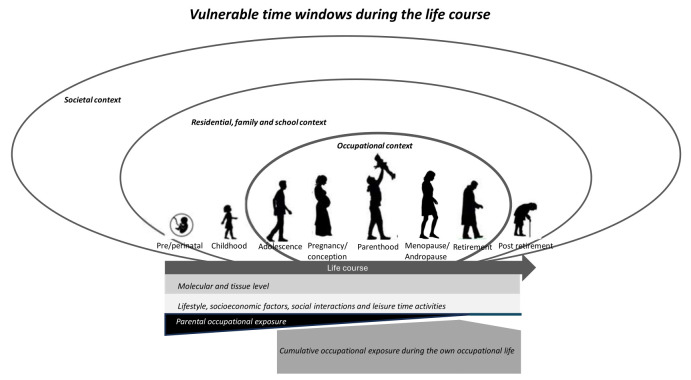
Vulnerable time windows during the life course

During the individual’s life course, several vulnerable time windows for the impact of a multitude of exposures that potentially harm, protect or promote health, eg, occupational, environmental and social, can be distinguished. The (combinations of) exposures may operate in different life stages and contexts and – directly or indirectly via intergenerational transmission – contribute to health (figure 1). The individual may be particularly sensitive to harmful exposures or adverse experiences during developmental life stages, ie, pre/perinatal, childhood, adolescence, pregnancy and menopause/andropause. Other life stages may reflect vulnerable time windows due to a clustering of exposures, eg, work and family demands during parenthood, or an accumulation of exposures during the (working) life course at retirement and post-retirement age.

As illustrated in figure 1, occupational exposure(s) can be divided in exposure through the parents’ exposure (early in life) and an individuals’ own exposure (later in life). Already in the ***pre/perinatal life stage,*** occupational exposure starts through the intergenerational transmission of the parents’ occupational exposures. Current and bioaccumulated occupational exposure of chemicals and particles in the father at the time of conception can affect sperm quality. Together with the mother’s exposure to occupational exposures of chemicals and particles prior to conception – or chemicals, particles, physical factors, ergonomic load, organizational and (psycho-)social conditions at work during pregnancy – this may affect fetal development and later disease development during the child’s life course (11–15). During ***childhood***, the growing child is exposed to parental occupational exposure(s), directly through chemicals and particles in the work clothes and skin or indirectly through organizational and psychosocial factors in the work environment that may increase the risk for mental and physical health problems in parents, which in turn may affect their parental rearing quality (16, 17).

During ***adolescence*** *and early adulthood*, individuals usually encounter their first direct occupational exposures through their first (student) job or jobs. Already from this life stage, occupational exposures may accumulate during the (working) life course and may affect not only the active working life but also the post working life. Also important to note is that brain plasticity is not limited to childhood, adolescence or young adulthood as it persists throughout life. Some studies indicate that high physical and chemical exposure during this life stage, can increase the risk of disease later in life (18). A poor psychosocial school or work environment in younger years may also increase the risk of adverse labour market outcomes and mental health problems later in life (19, 20). In adulthood, men and women often start with (the planning of) family formation. Some occupational exposures affect fecundability, others can increase the risk of pregnancy-related disease, such as preeclampsia, hypertension or diabetes, or affect the offspring (21, 22). Chemicals, heat and stress-related exposures affect the ability to conceive. During ***pregnancy***, the bodily and mental systems are vulnerable with changes in the endocrine and inflammation response that can dysregulate the HPA-axis, resulting in a prolonged stress response. The placenta can filter out many hazards, but not all toxicants, such as methylmercury and arsenic (23, 24). Physical exposure, such as noise and vibration, but also shift and night work can affect the womb and cause fetal growth restriction, preterm birth, and hearing impairment (eg 12, 13, 25–27,). During ***parenthood***, occupational exposures may affect the parents’ (mental) health and work-family balance (28, 29). Many chemical and physical exposures have now manifested in disease, eg, allergy, asthma and musculoskeletal diseases (28). During ***menopause*** in women, with a drastic decrease in oestrogen, and the slow testosterone decline in men (sometimes referred to as ***andropause***), dysregulations of the hormone system may disrupt and affect the individual’s susceptibility for occupational exposures in a way similar to environmental exposures (30). Towards **retirement**, the total cumulative occupational exposure burden over the working life course and the current exposure will affect the ability to stay at work and in the labor market. ***Post retirement***, most direct occupational exposures have ceased, but others may have (bio-) accumulated over time and may cause health problems that manifest after retirement (31, 32).

Along with occupational exposures, a multitude of other exposures are present during the entire life course that may operate across in different contexts to contribute to health (see figure 1). For instance, chemical, physical and social stressors during the life course leave traces (‘memories’) on the ***molecular and tissue levels*** that may affect later life health (33). Epigenetic marks act as heritable memories in the cell as they respond to different endogenous and exogenous signals and can be propagated from one generation of cells to the next generation of cells (33). Next to the epigenetic marks, the ***social environment and social determinants of health*** during the life course, eg, socio-economic and lifestyle factors, social relationships, social cohesion and support, are known to impact health and add to the multitude of exposures to be examined, among others in conjunction with the environmental exposome (eg 34,). In ***residential, family and school contexts***, exposures such as air pollution, drinking water pollution, noise, artificial light at night, limited access to green space and crowding may play a role, as can adverse childhood experiences (eg 35, 36,). Moreover, on the overarching ***societal context***, legislations, labor market conditions, norms, values and cultural aspects may affect worker health (2, 37).

## Main knowledge gaps and challenges

Both conceptual and empirical challenges have to be tackled when conducting work environment and health research with an interdisciplinary life course perspective. On the conceptual level, different paradigms and nomenclature still exist in the various disciplines examining the impact of (occupational) exposures on later life health outcomes, which contributes to fragmented research and publication thereof in specialized journals. On the empirical level, questions arise such as: Is it feasible to examine mechanisms and pathways across different exposure levels considering a life course perspective? Is the follow-up duration of existing birth and other cohorts sufficient to address the dynamic interplay between the work environment and health? Are the multifaceted, constantly changing contexts captured? Effect sizes are often small on an individual level and statistical power decreases when several rare assumptions have to be fulfilled to examine clusters or combinations of exposures and contexts in relation to health outcomes.

Big data, interdisciplinary research protocols and innovative, advanced statistical models to capture the life course perspective are needed to proceed beyond the exposome studies that are currently being finalized within the EU Horizon 2020 exposome call (https://www.humanexposome.eu/). Moreover, a better understanding is needed of how occupational, environmental and social exposures affect individuals (i) in vulnerable time windows, eg, do exposures contribute to health advantages and/or disadvantages, and (ii) while transitioning between and within different life stages (38). Studies in different disciplines have focused on the childhood and retirement life stages, see eg, the research on the school-to-work transition or the work-to-retirement transition (39–41), but little is known about the menopause or andropause life stage. Last, rigorous examinations of different life course models (eg, sensitive periods) and exposure models (eg, current, first, last, peak, single, chronic or accumulated), and their impact on health are needed within and across the different vulnerable time windows and life stages as exposure-outcome relationships may differ and thus call for targeted (preventive) policies and practices (42–44).

## Interdisciplinary research opportunities

The challenges towards a better understanding of the complex and dynamic interplay between the work environment and health provide ample opportunities for rigorous, collaborative quantitative and in-depth qualitative life course research across different research strands. Researchers from different disciplines, such as occupational and environmental medicine, epidemiology, toxicology, heal6th science, sociology, psychology, demography, public (mental) health, and genetics to name a few, should not shy away from the complexity, but embrace the opportunity to use their knowledge and skills to collectively address relevant research questions.

Interdisciplinary research opportunities are already present today and will emerge even more in the years to come as more cohorts designed as birth cohorts or multi-generational cohorts mature (eg, LifelinesNext, 45). Researchers have or get access to (national) registers, databases with individual-level internal and external exposure information and neighbourhood-level exposure information or linkages of all these exposure and health data, allowing them to examine the impact of exposures in advanced causal models on later life health. To illustrate the value of and research opportunities with existing data, Ubalde-Lopez and colleagues (46) recently argued that parental work-related data collected in birth cohorts is a valuable yet underutilized resource that could be exploited more fruitfully in the collaboration between birth cohort research, occupational epidemiology and sociology. Having said that, the authors also refer to the possible constraints of eg, cross-national comparative research in terms of technical (ie, harmonization) and ethical challenges (46).

In conclusion, to move research on the work environment and health forward, we call for a more integrated, interdisciplinary approach that considers the timing and accumulation of occupational, environmental and social exposures over the life course.
